# Calpain-6, a Target Molecule of Glucocorticoids, Regulates Osteoclastic Bone Resorption via Cytoskeletal Organization and Microtubule Acetylation

**DOI:** 10.1002/jbmr.241

**Published:** 2010-09-02

**Authors:** Jung Min Hong, Steven L Teitelbaum, Tae-Ho Kim, F Patrick Ross, Shin-Yoon Kim, Hyun-Ju Kim

**Affiliations:** 1 Skeletal Diseases Genome Research Center, Kyungpook National University HospitalDaegu, Korea; 2 Department of Pathology and Immunology, Washington University School of MedicineSt Louis, MO, USA; 3 Department of Orthopedic Surgery, School of Medicine, Kyungpook National UniversityDaegu, Korea; 4 Department of Medicine, School of Medicine, Kyungpook National UniversityDaegu, Korea

**Keywords:** Capn6, OSTEOCLAST, CYTOSKELETON, MICROTUBULE, GLUCOCORTICOIDS

## Abstract

Glucocorticoids (GCs) inhibit the resorptive capacity of the osteoclast by disrupting its cytoskeleton. We find that calpain-6 (Capn6), a unique, nonproteolytic member of its family, is suppressed 12-fold by dexamethasone (DEX) in the bone-degrading cell. While Capn6 abundance parallels commitment of naive bone marrow macrophages (BMMs) to the osteoclast phenotype, its excess or deletion does not affect the cell's differentiation. On the other hand, Capn6 localizes to the sealing zone, and its overexpression promotes osteoclast spreading and large actin ring formation, eventuating in stimulated bone degradation. Conversely, Capn6 knockdown impairs cytoskeletal organization and the cell's resorptive capacity. Capn6 complexes with tubulin, and its absence inhibits microtubule acetylation and stability in the osteoclast. Knockdown of Capn6 also reduces β_3_-integrin subunit protein, another essential regulator of osteoclast cytoskeletal function. Reflecting Capn6 as a target molecule of GCs, microtubule stability and acetylation, as well as the expression of β_3_-integrin protein, are similarly suppressed in DEX-treated osteoclasts. Moreover, overexpression of Capn6 rescues GC-mediated disruption of osteoclast cytoskeleton. Thus Capn6 promotes cytoskeletal organization and microtubule stability in osteoclasts, and its inhibition may mediate the resorption-arresting properties of GCs. © 2011 American Society for Bone and Mineral Research.

## Introduction

Skeletal remodeling is an ever-occurring event in mammals and likely serves to replace effete bone with new. Hence agents such as glucocorticoids (GCs), which suppress remodeling, predispose to fracture.

Remodeling is initiated by bone resorption, which is tethered to bone formation. Thus inhibition of osteoclast activity is typically attended by parallel changes in the osteoblast. In this regard, the suppressed remodeling attending GC therapy is initiated by inhibited osteoclast function consequent to the cell's inability to organize its cytoskeleton. Since stimulated remodeling is the most successful approach to treating GC-induced osteoporosis,(1) identifying the means by which steroids blunt the osteoclast is therapeutically relevant.

The osteoclast cytoskeleton is unique, and its organization is initiated by contact with bone. In this circumstance, the cell polarizes and generates sealing zones or actin rings that isolate the resorptive microenvironment from the general extracellular space.(2) These polarizing events require an intact microtubule network, reflecting, in turn, appropriate tubulin acetylation.(3,4) For example, *Pyk2*^–/–^ osteoclasts are unable to produce actin rings as a result of impaired microtubule acetylation and stability, prompting increased bone density.(5) Osteoclast cytoskeletal organization also depends on ligation and activation of the α_v_β_3_-integrin.(6,7) Thus failure to express or activate α_v_β_3_-integrin compromises cell spreading and actin ring formation and consequently arrests resorption.(6,7)

We performed microarray analysis of GC-treated osteoclasts and found calpain-6 (Cpan6), a candidate cytoskeleton effector molecule. Classic calpains are calcium-dependent cysteine proteases(8–12) that participate in integrin-mediated cytoskeletal organization during migration. These enzymes cleave a number of cytoskeleton-associated proteins including talin, paxillin, tubulin, Src, Pyk2, and the β_3_-integrin subunit.(13–23) These calpain substrates are enriched in the osteoclast actin ring, and the classic calpains, Capn1 (µ-calpain) and Capn2 (m-calpain), regulate osteoclast motility and bone resorption by cleaving talin and Pyk2.(16) Unlike the classic calpains, Capn6 does not possess an active-site catalytic cysteine residue and therefore is likely not a functional protease.(24) Capn6, however, is biologically relevant because it is an endothelin-1 (ET-1) target involved in pharyngeal arch development.(25) Importantly, RNAi-mediated inactivation of Capn6 in transformed cells causes microtubule instability and enhances lamellipodium formation.

Whether Capn6 influences the osteoclast was heretofore unknown, but we found that it was expressed with differentiation and repressed by dexamethasone (DEX). Furthermore, Capn6 overexpression in the resorptive polykaryon accelerates microtubule acetylation and cytoskeletal organization. Conversely, Capn6 knockdown arrests microtubule acetylation and actin ring formation as well as diminishing β_3_-integrin protein. Similarly, DEX destabilizes microtubules and reduces β_3_-integrin protein expression. Capn6 therefore regulates microtubule- and integrin-associated organization of the cytoskeleton in osteoclasts, and its inhibition may be a means by which GCs suppress bone remodeling.

## Materials and Methods

### Macrophage isolation and osteoclast culture

Primary bone marrow–derived macrophages (BMMs) were prepared as described previously.(26) Briefly, the marrow was extracted from the tibias and femurs of 6- to 8-week-old C57/BL6 mice with α-minimal essential medium (α-MEM) and cultured for 3 days with α-MEM containing 10% fetal bovine serum (FBS) and 1/10 volume of CMG 14-12 culture supernatant.(27) The cells were lifted with 1× trypsin/EDTA, plated in a 96-well culture plate at a density of 5 × 10^3^ cells/well, and cultured in α-MEM containing 10% FBS in the presence of 100 ng/mL of receptor activator of nuclear factor κB ligand (RANKL) and 10 ng/mL of macrophage colony-stimulating factor (M-CSF). Cultured cells were fixed in 10% formaldehyde for 10 minutes and then 12.5 mM citrate buffer containing acetone (65%) and formalin (2.96%) for 1 minute. Osteoclasts were stained for tartrate-resistant acid phosphatase (TRAP) activity. For DEX treatment, BMMs were cultured in M-CSF and RANKL for 3 days to commit them to the osteoclast phenotype. The cells then were treated with vehicle or DEX (100 nM) for 2 days in the presence of RANKL and M-CSF.

### RT-PCR

Total RNA (1 µg) extracted from cultured cells was used as a template for cDNA synthesis. Primers were synthesized on the basis of the reported mouse cDNA sequence. The following primers were used for *Capn1* (sense, 5′-CGAGCCCAACAAAGAAGG-3′; antisense, 5′-CGCACAAGACAGCACACAA-3′), *Capn2* (sense, 5′-ACATCCACCTCGGCAAAA-3′; antisense, 5′-CCACTCCCATCTTCATCCA-3′), *Capn5* (sense, 5′-GCCATTCCAACTCCAAAAA-3′; antisense, 5′-GCCCATCTTCTCCCTCTCAC-3′), *Capn6* (sense, 5′-TCTTCTCATTCTCCACCTCCA-3′; antisense, 5′-GCCACTCCATTCCTGTCTTC-3′), *β*_*3*_*-integrin* (sense, 5′-TTACCCCGTGGACATCTACTA-3′; antisense, 5′-AGTCTTCCATCCAGGGCAATA-3′), *Atp6V0d2* (sense, 5′- TAGCCAAGTGTCACCCACTG-3′; antisense, 5′- TGATGATTAAAGCGCGTCTG-3′), *DC-STAMP* (sense, 5′- CGTGGAGAGAAGCAAGGAAC-3′; antisense, 5′-CAGCCTTGCAAACTCAAACA-3′), *TRAP* (sense, 5′-ACAGCCCCCCACTCCCACCCT-3′; antisense, 5′-TCAGGGTCTGGGTCTCCTTGG-3′), *MMP9* (sense, 5′-CCTGTGTGTTCCCGTTCATCT-3′; antisense, 5′-CGCTGGAATGATCTAAGCCCA-3′), *cathepsin K* (sense, 5′-GGAAGAAGACTCACCAGAAGC-3′; antisense, 5′-GTCATATAGCCGCCTCCACAG-3′), and *GAPDH* (sense, 5′-ACTTTGTCAAGCTCATTTCC-3′; antisense, 5′-TGCAGCGAACTTTATTGATG-3′). Amplification was conducted for 22 to 31 cycles, each of 94°C for 30 seconds, 55°C for 30 seconds, and 72°C for 30 seconds. Ten microliters of each reaction mixture was analyzed by 1.5% agarose gel electrophoresis.

### Retroviral transduction

Capn6 was cloned into pMX retroviral vector containing Flag and transfected into Plat-E packing cells (a gift from S Takeshita) using Transfectol (Gene Choice, Frederick, MD, USA). Virus was collected from cultured medium 24 to 48 hours after transfection. BMMs were transduced with virus for 24 hours in the presence of 4 µg/mL of Polybrene (Sigma-Aldrich, St Louis, MO, USA). Cells then were selected with 1 µg/mL of blasticidin (Calbiochem, San Diego, CA, USA) for 3 days.

### Lentiviral transduction of *Capn6* shRNA

Oligonucleotides encoding a target site for Capn6 and loop RNA were cloned into lentiviral vector. The lentiviruses encoding luciferase shRNA (*Luc*-sh) were used as a control. The sense sequences for *Capn6*-sh1 and *Capn6*-sh2 were 5′-GGACCACTGACATTCCTATTA-3′ and 5′-GGTTCCGTCTTCACCATCTGTA-3′, respectively. Recombinant lentiviral production was carried out according to the manufacturer's instructions (Invitrogen, Carlsbad, CA, USA). Briefly, the 293T producer cell line was cotransfected with the expression constructs and packaging mixture. The viral supernatant was harvested after 48 hours. BMMs were transduced with viral supernatant plus 10 µg/mL of protamine sulfate (Sigma-Aldrich) for 24 hours. Cells then were selected in the presence of 4 µg/mL of puromycin for 5 days.

### Western blot analysis and immunoprecipitation

Cells were harvested in lysis buffer (10 mM Tris, pH 7.4, 150 mM NaCl, 1% NP-40, 1 mM EDTA, and 10% glycerol) containing protease and phosphatase inhibitor. After incubation on ice for 10 minutes, the cell lysates were clarified by centrifugation at 14,000 rpm for 30 minutes. The protein concentration was measured with a Bicinchoninic Acid Kit (Pierce, Rockford, IL, USA), and an equal amount of each lysate was separated on an SDS-polyacrylamide gel. After the proteins were transferred to a polyvinylidene fluoride (PVDF) membrane (Millipore, Billerica, MA, USA), the membrane was blocked and incubated with primary antibodies. Monoclonal antibodies to FLAG M2 and acetylated tubulin (clone 6-11B-1) were obtained from Sigma-Aldrich. Polyclonal antibodies to Capn6 were from Abcam (Cambridge, MA, USA) or Santa Cruz Biotechnology (Santa Cruz, CA, USA). Monoclonal antibody to α-tubulin was from Abcam. All antibodies were diluted 1:1000. Proteins were visualized using an appropriate secondary antibody. This was followed by an application of enhanced chemiluminescence reagents.

For the immunoprecipitation, cells were harvested with lysis buffer (10 mM Tris, pH 7.4, 150 mM NaCl, 1% NP-40, 1 mM EDTA, and 10% glycerol) containing protease and phosphatase inhibitors. The lysates were immunoprecipitated with anti-α-tubulin antibody followed by incubation with protein A/G-Sepharose beads (Pierce) at 4°C and analysis by immunoblotting.

### Actin ring formation and bone resorption assay

BMMs were cultured on bone with RANKL (100 ng/mL) and M-CSF (10 ng/mL). Cells were fixed in 4% paraformaldehyde and permeabilized in 0.1% Triton X-100. After washing with PBS, cells were stained for actin with 10 µg/mL of Tetramethyl Rhodamine Isothiocyanate (TRITC)-conjugated phalloidin (Sigma-Aldrich). For the staining of resorption pits, cells were removed from bone slices with mechanical agitation. Bone slices were incubated with 20 µg/mL of peroxidase-conjugated wheat germ agglutinin for 45 minutes. After washing with PBS, 3,3′-diaminobenzidine (Sigma-Aldrich) was added onto the bone slices. To quantify the osteoclast bone resorption, the level of type I collagen fragments (CTX-1) was assayed using CrossLaps ELISA (Nordic Bioscience Diagnostics, Herlev Denmark), as described previously.(26)

### Immunofluorescence

Cells were fixed with 4% paraformaldehyde for 20 minutes and permeabilized with 0.1% Triton X-100, followed by blocking with 0.2% bovine serum albumin (BSA) for 10 minutes. Specific proteins were labeled with the following primary antibodies: the goat polyclonal anti-Capn6 antibody (diluted 1:50; Santa Cruz), monoclonal anti-FLAG M2 and anti-acetylated tubulin antibodies (diluted 1:100; Sigma), and anti-α-tubulin antibody (diluted 1:100; Abcam). After washing with PBS, secondary antibody was added for 1 hour at room temperature and then washed with PBS three times. Samples were mounted with 90% glycerol in PBS and observed using a confocal microscope (LSM, Carl Zeiss, Jena, Germany). All images from the same set of experiments were taken without changing the camera settings.

### Statistics

All data are presented as mean ± SD. Statistical significance was determined by two-tailed Student's *t* test.

## Results

### Capn6 is a GC target upregulated during osteoclastogenesis

**Fig. 1:**
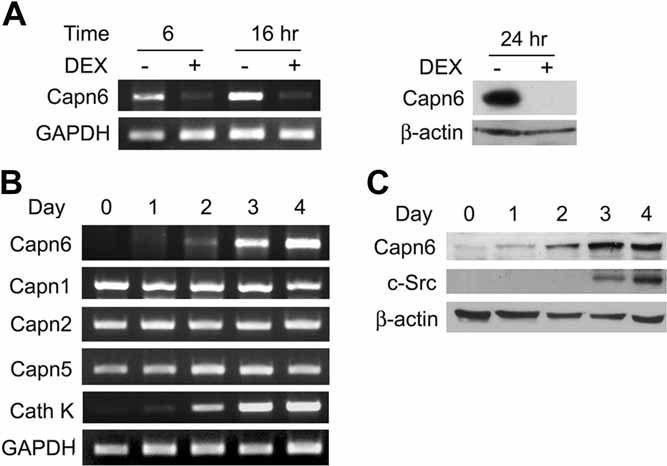
Expression of Capn6 is regulated by dexamethasone and enhanced during osteoclast development. (*A*) BMMs were cultured in M-CSF and RANKL for 3 days to commit them to the osteoclast phenotype. The cells then were maintained without or with DEX (100 nM) for the indicated times. Capn6 expression was determined by RT-PCR (*left*) or immunoblotting (*right*). (*B*, *C*) BMMs were cultured in M-CSF (20 ng/mL) and RANKL (100 ng/mL) with time. (*B*) Calpain family member mRNA was determined by RT-PCR. (*C*) Capn6 expression was analyzed by immunoblotting. *Cathepsin K* (*CathK*) or *c-Src* serves as a positive control for osteoclastogenesis and *GAPDH* or *β-actin* as loading control.

To identify GC targets, we committed precursors to the osteoclast phenotype by 3 days of exposure to M-CSF and RANKL. The cells were treated with vehicle or DEX for 16 hours in osteoclastogenic medium containing charcoal-stripped FBS. Microarray analysis demonstrates that Capn6 is decreased 12.8-fold in osteoclasts exposed to the steroid (data not shown). Confirming the relevance of this observation, *Capn6* mRNA and protein are reduced by DEX treatment (Fig. 1*A*).

To determine the expression pattern of calpain family proteins during osteoclast formation, BMMs were exposed to osteoclastogenic cytokines. While the classic calpains (Capn1 and -2) and *Capn5* mRNAs are expressed constitutively but not induced, *Capn6* mRNA increases as the cells differentiate (Fig. 1*B*). Similarly, Capn6 protein accumulates incrementally throughout the process (Fig. 1*C*).

### Capn6 modulates osteoclast spreading

**Fig. 2:**
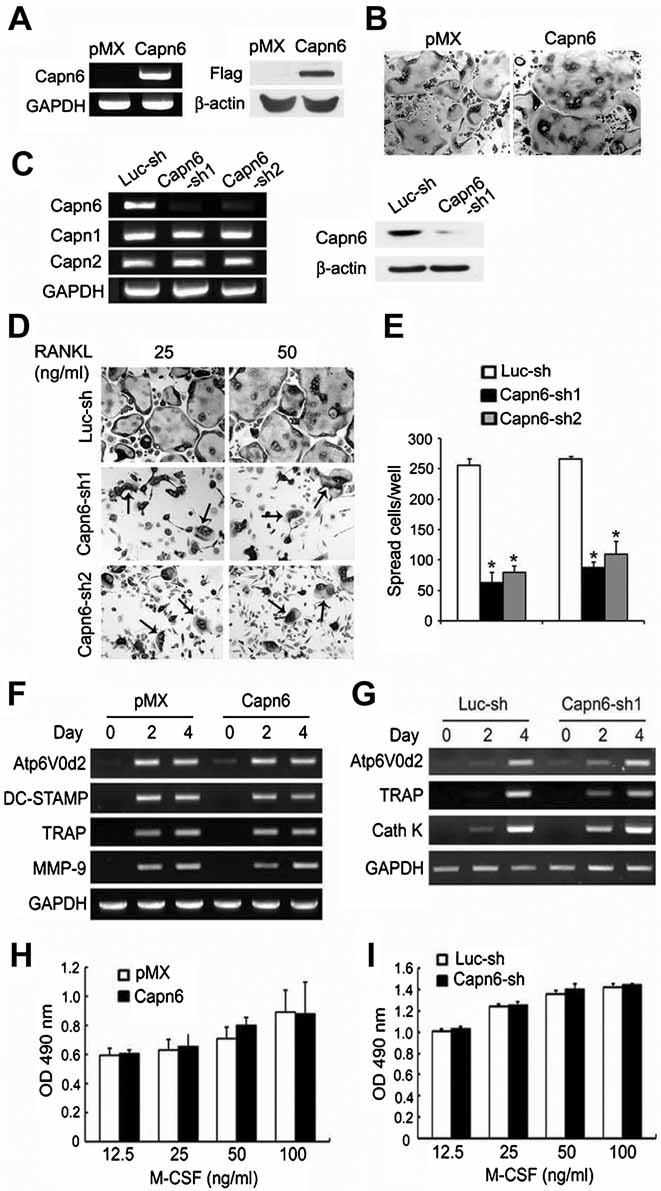
Capn6 regulates cell spreading. (*A*, *B*) BMMs were transduced with pMX vector containing Flag or Flag-Capn6. (*A*) Capn6 expression was analyzed by RT-PCR (*left*) or immunoblotting (*right*). (*B*) Osteoclasts were generated with 25 ng/mL of RANKL and M-CSF and stained for TRAP activity after 4 days. (*C*–*E*) BMMs were transduced with *Luc*-sh- (control) or *Capn6*-shRNA (*Capn6*-sh1 and *Capn6*-sh2) and cultured in M-CSF and RANKL. (*C*) *Capn6* and classic *calpain* mRNAs were determined by RT-PCR (*left*) or Capn6 protein expression by immunoblotting (*right*). (*D*) Cells were stained for TRAP activity after 5 days. Arrows indicate nonspread TRAP^+^ osteoclasts. (*E*) Statistical analysis of characteristic spread TRAP^+^ multinucleated cells per well. *p < 0.001. (*F*) BMMs were transduced with pMX bearing Flag or Flag-Capn6 and cultured for 4 days with M-CSF alone (day 0) or M-CSF and RANKL (days 2 and 4). RT-RCR was performed for the expression of fusion or osteoclastogenic markers. (*G*) BMMs were transduced with *Luc*-sh or *Capn6*-sh1 and cultured as described in panel *F*. RT-PCR analysis was performed. *GAPDH* serves as loading control. (*H*) BMMs were transduced with pMX bearing Flag or Flag-Capn6. Equal numbers of BMMs were cultured with the indicated concentrations of M-CSF for 3 days. 3-(4,5-dimethylthiazol-2-yl)-5-(3-carboxymethoxyphenyl)-2-(4-sulfophenyl)-2H-tetrazolium (MTS) assay was performed by measuring optical density (OD) values at 490 nm. (*I*) Equal numbers of BMMs transduced with *Luc*-sh or *Capn6*-sh1 were cultured with the indicated concentrations of M-CSF. After 3 days, MTS assay was performed.

To investigate its role in osteoclast development, we retrovirally transduced wild-type (WT) BMMs with Capn6 or empty vector (Fig. 2*A*). The cells were cultured in M-CSF and RANKL, and osteoclasts were identified by TRAP activity. Capn6 overexpression yields osteoclasts that are spread and larger than their vector-transduced counterparts (Fig. 2*B*).

We explored the role of endogenous Capn6 in osteoclast development by transducing BMMs with lentivirus carrying *Capn6* small hairpin RNAs (shRNA) (denoted as *Capn6*-sh1 and *Capn6*-sh2) or control *luciferase* shRNA (*Luc*-sh). The shRNAs substantially reduce *Capn6* mRNA and/or protein (Fig. 2*C*). Establishing specificity, *Capn6* knockdown does not alter the abundance of classic *calpain* mRNA.

We next determined the impact of altered Capn6 expression on osteoclast morphology. Thus *Luc*-sh-, *Capn6*-sh1-, and *Capn6*-sh2-bearing BMMs were cultured with M-CSF and 25 or 50 ng/mL of RANKL for 5 days and stained for TRAP activity. Whereas *Luc*-sh transductants form characteristic well-spread osteoclasts, *Capn6*-shRNA inhibits spreading, yielding TRAP expressing polykaryons with a “crenated” appearance similar to those with established cytoskeletal defects (Fig. 2*D, E*).

To determine if morphologic changes induced by Capn6 excess or deficiency reflect altered differentiation and/or fusion of osteoclast precursors, we assessed mRNA of relevant proteins during the osteoclastogenic process. Neither increased Capn6 nor its knockdown has an impact on gene expression of the fusion marker DC-STAMP or a variety of indicators of osteoclast differentiation (Fig. 2*F, G*). Furthermore, overexpression or knockdown of Capn6 does not have an impact on the number of viable osteoclast precursors exposed to various concentrations of M-CSF (Fig. 2*H, I*).

### Capn6 modulates cytoskeletal organization

**Fig. 3:**
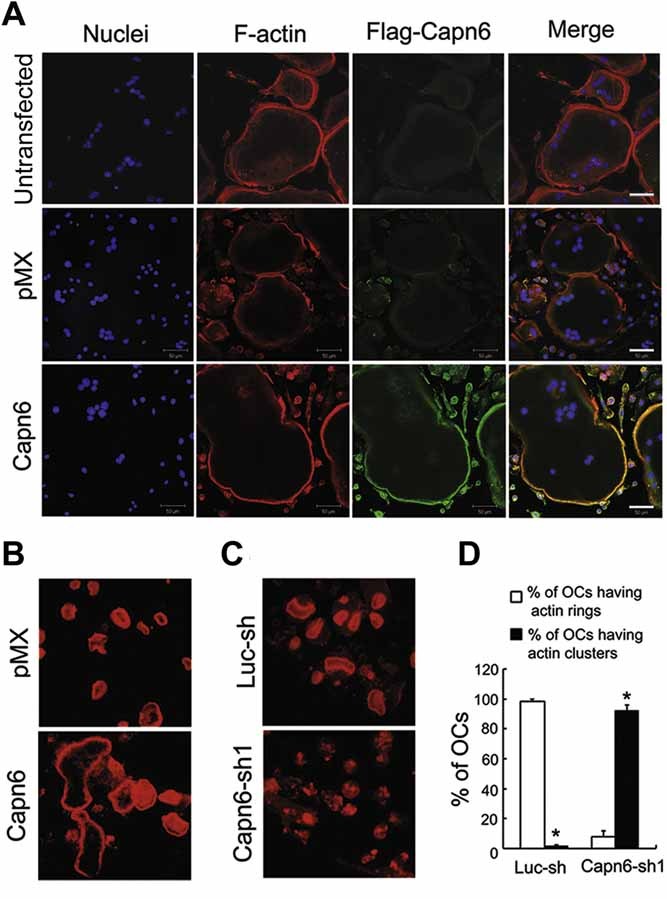
Alteration of Capn6 expression regulates actin ring formation. BMMs were transduced with pMX vector containing Flag or Flag-Capn6 and cultured on coverslips (*A*) or bone slices (*B*) for 5 days with M-CSF and RANKL. (*A*) Nuclei, F-actin, and Flag-Capn6 were stained with Hoechst (*blue*), TRITC-conjugated phalloidin (*red*), and anti-flag antibody (*green*), respectively, and detected by confocal microscopy. Untransfected cells were stained as a control. Scale bars = 50 µm. (*B*) Actin ring formation was examined by immunofluorescence after TRITC-phalloidin staining. (*C*, *D*) BMMs transduced with *Luc*-sh or *Capn*6-sh1 were seeded on bone and exposed to M-CSF and RANKL for 6 days. (*C*) The cells were fixed and stained with TRITC-phalloidin to visualize actin rings. (*D*) Quantification of percentage of osteoclasts containing actin rings (*white bars*) or clusters (*black bars*). *p < 0.001.

Given that altered expression of Capn6 does not affect osteoclast fusion or differentiation, we focused on function, particularly in the context of the cytoskeleton. To determine if Capn6 associates with actin rings of bone-resorptive cells, osteoclasts were generated, on coverslips, from BMMs transduced with pMX or Flag-tagged Capn6. Immunostaining with anti-actin and anti-Flag antibodies reveals that Capn6 localizes to the actin ring, suggesting that it may participate in cytoskeletal organization (Fig. 3*A*). To confirm that such is the case, we generated osteoclasts on bone slices in M-CSF and RANKL for 5 days and stained the actin cytoskeleton with TRITC-conjugated phalloidin. Reflecting enhanced spreading, osteoclasts transduced with Capn6 contain larger actin rings than control osteoclasts (Fig. 3*B*). Further establishing that the enzyme organizes the cytoskeleton, Capn6-silenced osteoclasts failed to generate rings from actin clusters (Fig. 3*C, D*). Thus Capn6 excess enhances cytoskeletal organization, whereas its deficiency is inhibitory.

### Capn6 regulates microtubule distribution and acetylation

**Fig. 4:**
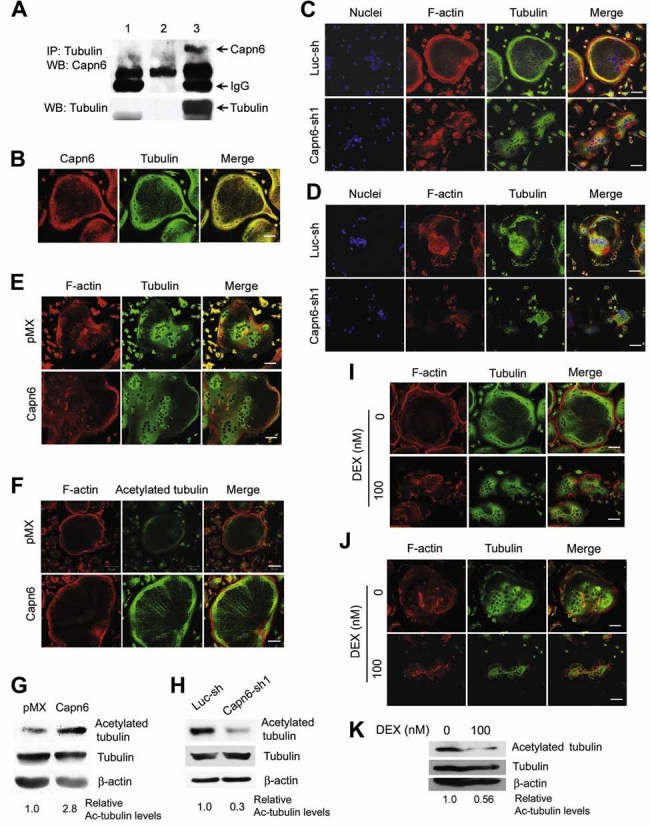
Capn6 and DEX modulate osteoclast microtubule distribution and acetylation. (*A*, *B*) Endogenous Capn6 colocalizes to the microtubule. BMMs were cultured in M-CSF and RANKL for 4 days. (*A*) Osteoclast lysates were immunoprecipitated with anti-tubulin antibody and subjected to immunoblotting with the indicated antibodies. (*Lane 1*) Protein beads with anti-tubulin Ab but without cell extract (negative control). (*Lane 2*) Protein beads with cell extract but without Ab (negative control). (*Lane 3*) Protein beads with cell extract and Ab. (*B*) Osteoclasts were stained with anti-Capn6 (*red*) and anti-tubulin (*green*) antibody. (*C*, *D*) BMMs were transduced with *Luc*-sh or *Capn6*-sh1 and cultured on coverslips in M-CSF and RANKL for 5 days. (*C*) Cells were stained with Hoechst (*blue*), TRITC-conjugated phalloidin (*red*), and anti-tubulin (*green*) antibody. (*D*) Osteoclasts were treated with 2 µM nocodazole for 1 hour, fixed, and labeled. (*E*–*G*) BMMs were transduced with pMX containing Flag or Flag-Capn6. Osteoclasts were generated on coverslips. (*E*) The cells were fixed and labeled. (*F*) Osteoclasts were stained with TRITC-conjugated phalloidin (*red*) and anti-acetylated tubulin (*green*) antibody. (*G*) Osteoclast lysates were immunoblotted with the indicated antibodies. The ratio of acetylated tubulin to β-actin was determined by densitometry. (*H*) BMMs transduced with *Luc*-sh or *Capn6*-sh1 were cultured in M-CSF and RANKL. Osteoclast lysates were immunoblotted with the indicated antibodies. (*I–K*) Dexamethasone inhibits osteoclast microtubule stability. BMMs were cultured in M-CSF and RANKL for 3 days to commit them to the osteoclast phenotype. The cells then were maintained without or with DEX (100 nM) for 2 days. (*I*) Osteoclasts were labeled with TRITC-phalloidin and anti-tubulin antibodies. (*J*) Mature osteoclasts were treated with 2 µM nocodazole for 1 hour, fixed, and labeled. (*K*) Osteoclast lysates were immunoblotted with the indicated antibodies. The ratio of acetylated tubulin to β-actin was determined by densitometry. All scale bars indicate 50 µm.

During bone resorption, actin clusters mature into rings, a process that depends on functioning microtubules.(28) To determine if endogenous Capn6 interacts with microtubules, tubulin was precipitated from mature osteoclasts. Immunoblotting with anti-Capn6 antibody documented that Capn6 associates with microtubules in osteoclasts (Fig. 4*A*). In confirmation of these data, immunostaining demonstrated that Capn6 localizes to the microtubule network (Fig. 4*B*).

To investigate the functional implications of this association, we assessed the impact of Capn6 knockdown on microtubule organization. As seen in Fig. 4*C*, cells transduced with *Luc*-sh contain radial microtubules enriched in the vicinity of the actin ring. In contrast, the microtubule network of *Capn6*-sh-bearing osteoclasts is irregular and not juxtaposed to the cell's periphery. Similarly, Capn6 knockdown osteoclasts contain fewer nocodazole-resistant stable microtubules than cells transduced with *Luc*-sh (Fig. 4*D*). Conversely, nocodazole-resistant stable microtubules are enhanced in Capn6-overexpressing osteoclasts (Fig. 4*E*).

Microtubules in mature osteoclast are acetylated, which, in turn, maintains their stability.(28) Because Capn6 overexpression enhances microtubule acetylation and stability in HeLa cells,(25) we asked whether the same holds in osteoclasts. In fact, Capn6-transduced osteoclasts contain an abundance of acetylated tubulin relative to control (Fig. 4*F*). This morphologic observation is confirmed by immunoblotting total tubulin and its acetylated form in the same cells (Fig. 4*G*). Conversely, tubulin acetylation in Capn6 knockdown osteoclasts is reduced substantially (Fig. 4*H*).

### GCs inhibit microtubule stability and acetylation in osteoclasts

Because GCs suppress osteoclast spreading and actin ring formation,(29) we examined the effects of the steroid on osteoclast microtubules. To this end, we committed BMMs to the osteoclast phenotype in the presence of M-CSF and RANKL for 3 days. The cells were treated with vehicle or DEX for 2 days in osteoclastogenic medium. While the control cells contain characteristic radial microtubules, similar to the Capn6 knockdown osteoclasts, the network of those treated with DEX was irregular (Fig. 4*I*). We also found that DEX-treated osteoclasts possess few nocodazole-resistant microtubules (Fig. 4*J*). This DEX-mediated microtubule instability is confirmed by immunoblots showing the reduced acetylated tubulin in steroid-exposed osteoclasts (Fig. 4*K*).

### Capn6 and GCs regulate β_3_-integrin expression

**Fig. 5:**
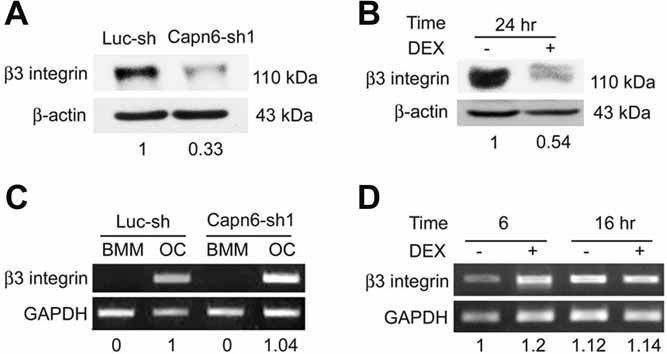
Capn6 knockdown or dexamethasone suppresses β_3_-integrin protein expression. (*A*) BMMs were transduced with *Luc*-sh or *Capn6*-sh1 and cultured in M-CSF and RANKL for 4 days. Lysates were immunoblotted for β_3_-integrin subunit. Actin serves as loading control. (*B*) BMMs were cultured in M-CSF and RANKL for 3 days. DEX (100 nM) or vehicle was added for an additional 24 hours. Lysates were immunoblotted for β_3_-integrin subunit. Actin serves as loading control. (*C*) *Luc*-sh- or *Capn6*-sh1-transduced BMMs were cultured in M-CSF (BMMs) or M-CSF and RANKL (OCs) for 4 days. *β_3_-Integrin* mRNA expression was determined by RT-PCR. *GAPDH* serves as loading control. (*D*) BMMs were cultured for 3 days in M-CSF and RANKL. DEX (100 nM) was added for an additional 6 or 16 hrs. *β_3_-Integrin* mRNA expression was determined by RT-PCR. *GAPDH* serves as loading control. Relative β_3_-integrin expression was determined by densitometry.

The α_v_β_3_-integrin is key to organizing the osteoclast cytoskeleton. In fact, both Capn6- and β_3_-integrin-deficient osteoclasts enjoy a similar phenotype in that they fail to spread, yielding the common “crenated” appearance induced by deletion of a variety of cytoskeleton-regulating molecules. Suggesting that α_v_β_3_-integrin mediates Capn6's effects on the osteoclast, expression of β_3_-integrin protein is reduced in *Capn6*-sh1-transduced cells (Fig. 5*A*).

DEX decreases the expression of β_3_-integrin protein (Fig. 5*B*). Alternatively, β_*3*_*-integrin* mRNA is not altered by either Capn6 knockdown or DEX, indicating a posttranscriptional event (Fig. 5*C, D*).

### Capn6 regulates bone resorption

**Fig. 6:**
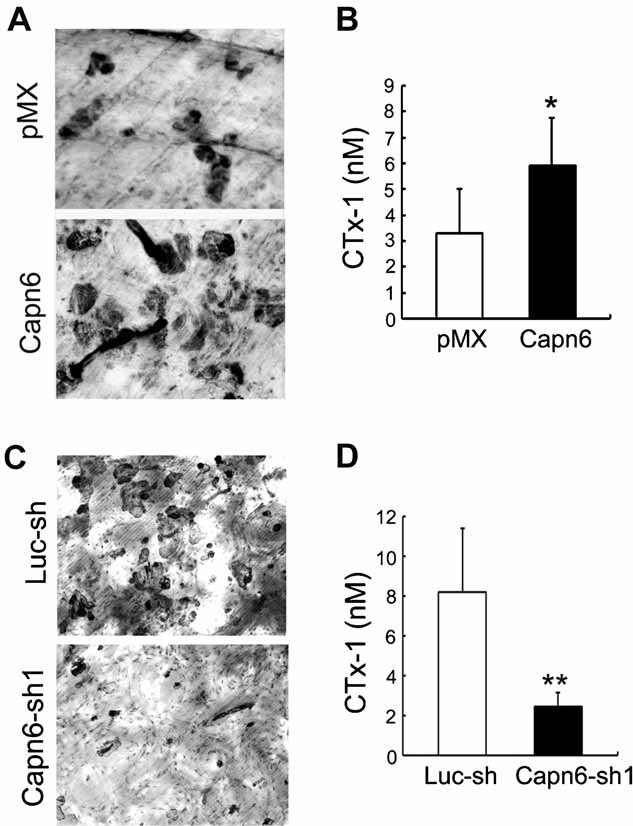
Capn6 modulates bone-resorptive activity. (*A*, *B*) BMMs were transduced with pMX containing Flag or Flag-Capn6 and cultured on bone slices with M-CSF and RANKL. (*A*) After 5 days, osteoclasts were removed, and resorption pits were visualized with peroxidase-conjugated wheat germ agglutinin. (*B*) Medium was collected and assayed for CTX-I content by ELISA. *p < 0.05. (*C*, *D*) BMMs transduced with *Luc*-sh or *Capn6*-sh1 were cultured on bone in the presence of M-CSF and RANKL for 6 days. (*C*) Lacuna formation was visualized. (*D*) Total bone resorption was determined by CTX-I ELISA of culture medium. **p < 0.001.

Cytoskeletal alteration in the osteoclast is typically mirrored by its resorptive capacity, and we asked whether the same obtains in the context of Capn6. Thus we generated control and Capn6-overexpressing or -deleted osteoclasts on bone. Reflecting their “superspread” appearance, bone degradation by Capn6-transduced polykaryons is enhanced, as evidenced by abundant, large pits and, most importantly, release of collagen type I degradation products (Fig. 6*A, B*). In contrast, the same assays established that Capn6-silenced cells inefficiently resorb bone (Fig. 6*C, D*).

### Capn6 overexpression reverses GC-mediated disruption of cytoskeletal organization

**Fig. 7:**
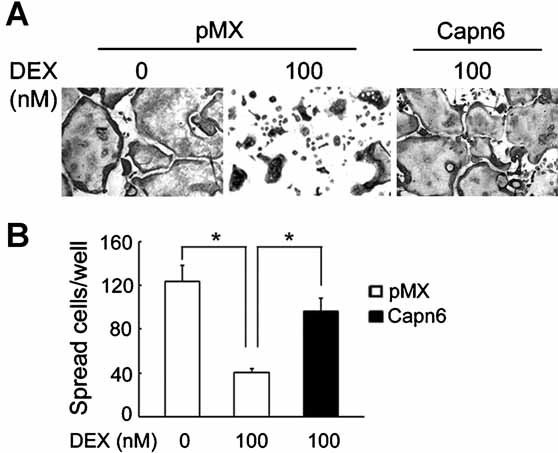
Overexpression of Capn6 rescues the inhibitory effect of dexamethasone on osteoclast cytoskeleton. BMMs were transduced with pMX vector containing Flag or Flag-Capn6 and cultured in M-CSF and RANKL for 3 days to commit them to the osteoclast phenotype. The cells then were maintained without or with DEX (100 nM) for 2 days. (*A*) The cells were stained for TRAP activity. (*B*) Statistical analysis of characteristic spread TRAP^+^ multinucleated cells per well. *p < 0.005.

To determine whether ectopic expression of Capn6 rescues the inhibitory effect of GCs on the osteoclast cytoskeleton, we retrovirally transduced BMMs with pMX empty vector or Capn6 and committed them to the osteoclast phenotype in M-CSF and RANKL for 3 days. The cells then were treated with vehicle or DEX for 2 days in osteoclastogenic medium and stained for TRAP activity. Capn6 overexpression rescues DEX-mediated disruption of the osteoclast cytoskeleton (Fig. 7).

## Discussion

GC treatment is the second most common cause of osteoporosis, reflecting, in part, direct inhibition of the osteoblast. GCs, however, potently inhibit osteoclasts, and their chronic use is characterized by suppressed bone turnover. Thus DEX also blunts osteogenesis by arresting the initiating event of remodeling, namely, osteoclast activation. The consequences of GC-attenuated remodeling are not only osteoporosis but also compromised bone quality, eventuating in diminished mechanical stability per unit of skeletal mass.(30) Steroid-suppressed resorption, therefore, likely predisposes to fracture.

Bone degradation is modulated by altering the abundance or matrix-degrading capacity of osteoclasts. While GCs acutely blunt M-CSF-stimulated proliferation of BMMs, they also prolong longevity of the differentiated polykaryon.(29,31) These compensating properties in combination yield no change in osteoclast number. On the other hand, DEX disrupts the osteoclast cytoskeleton and in so doing attenuates the cell's bone-resorptive capacity.(29) Given the clinical implications of steroid-suppressed osteoclast function, we turned to the means by which DEX exerts its anti-resorptive effects.

Our efforts were initiated by microarray analysis of GC- and vehicle-treated osteoclasts, wherein 387 genes are suppressed, but most prominently *Capn6*. Unlike the classic family members, *Capn1* (µ-calpain) and *Capn2* (m-calpain), which are constitutive and unchanged in osteoclasts and their undifferentiated precursors, *Capn6* is substantially upregulated during the osteoclastogenic process. Thus Capn6 presents as a candidate mediator of osteoclast function and a GC target in the mature cell.

Unlike the suppressive effects of GCs on BMMs, Capn6 abundance or deficiency does not alter the numbers of viable osteoclast precursors. Thus the Capn6 is unlikely to modulate DEX's anti-proliferative effects on BMMs. Like the steroid, however, Capn6 does not retard differentiation but regulates the capacity of osteoclasts to spread, a manifestation of actin organization. Capn6 overexpression promotes a “superspread” appearance of the polykaryon, with enlarged actin rings and enhanced capacity to resorb bone. A reciprocal phenotype is achieved by silencing the *Capn6* gene, yielding a dominance of nonspread hyporesorptive cells that lack actin rings. These observations establish that Capn6 is a regulator of the osteoclast cytoskeleton, which is in keeping with its localization within the sealing zone. Furthermore, Capn6-deficient osteoclasts resemble those treated with GCs, buttressing the hypothesis that the steroid exerts its effects via Capn6 inhibition.

While the osteoclast cytoskeleton is unique in structure, like that of all cells, it remodels with function. Thus actin is diffusely distributed throughout the cytoplasm in the nonresorptive, motile state and polarizes into the sealing zone during bone degradation. The generation of large actin rings and lacunae by osteoclasts overexpressing Capn6 and the clustered actin and scattered small pits appearing in the face of its suppression confirm that Capn6 modulates the cell's cytoskeleton *pari passu* with resorptive function.

Formation of the actin ring occurs under the aegis of the α_v_β_3_-integrin heterodimer when the cell contacts bone. The liganded integrin initiates a signaling pathway involving c-Src, Syk, ITAM-adaptor proteins, SLP-76, and Vav-3 that induces GTPases such as Rac to transit from their inactive GDP-bound state to their active GTP-bound conformation. The suppressive effect of *Capn6* deletion on β_3_-integrin subunit expression therefore likely contributes to the associated cytoskeletal disorganization. Recent evidence indicates, however, that integrin-mediated actin ring formation may involve an additional major component, namely, microtubules.

Microtubules are key regulators of the mitotic machinery governing spindle position and polarity. In addition, they participate in actin filament distribution within the contractile ring whose action completes cell division. The functionality of microtubules depends on cycling between their acetylated, stable and deacetylated, destabilized states. In keeping with a tethering of integrin- and microtubule-mediated actin remodeling, α_v_β_3_-integrin occupancy acetylates tubulin.(32) Deletion of the integrin's target, *Pyk2*, exerts the opposite effect and yields abnormal actin rings, as occurs in α_v_β_3_-integrin-deficient polykaryons. Finally, failure to acetylate or deacetylate tubulin enhances bone mass presumably owing to osteoclast dysfunction.(5,33)

We found that Capn6, which is inhibited by GCs, binds to and acetylates osteoclast tubulin, thereby stabilizing the cell's microtubules. These stabilized microtubules are juxtaposed on the actin ring and radiate toward the cell's center. Capn6 overexpression accentuates this radial distribution of microtubules. Alternatively, downregulation of the Capn6 removes deacetylated tubulin from the periphery and alters its filamentous appearance. The acetylation and deacetylation induced by the respective increase and decrease in Capn6 are mirrored by the cell's resorptive activity.

Maintenance of tubulin's acetylated state in the osteoclast is proposed to involve RhoA activation, which, in turn, blunts histone deacetylase 6 (HDAC6). GCs, however, suppress Capn6 but inhibit RhoA activation. Whereas HDAC6 inhibition may participate in microtubule-mediated osteoclast activity, the fact that deletion of the deacetylase in mice eventuates in a mild bone phenotype(33) indicates that other mechanisms of stabilization exist.

Our studies and others are consistent with a dynamic model of osteoclast function in which the acetylated state of tubulin plays a central role. During the bone-degrading phase of the resorptive cycle, wherein polarized actin ring formation is required, microtubules must be acetylated and stabilized. On cessation of matrix resorption and induction of motility, actin transits into its clustered conformation, and microtubules are deacetylated. The similarity of DEX-treated osteoclasts and those with deacetylaed tubulin owing to Capn6 downregulation suggests that inhibition of the Capn6 may be a component of the resorption-inhibiting properties of the steroid.
